# The Hospital. Nursing Section

**Published:** 1901-11-09

**Authors:** 


### The Hospital.

### Nursing Section.

Contributions for this Section of "THE HOSPITAL" should be addressed to the EDITOR, "THE HOSPITAL" NURSING SECTION, 28 & 29 Southampton Street, Strand, London, W.C.

No. 789.—VOL. XXXI. *SATURDAY, NOVEMBER* 9, 1901.

### Notes on News from the Nursing World.

### "NO WOMAN TO ATTEND UPON HIM."

THE immediate result of the speech of Mr. Chamberlain on behalf of the Scottish Branch of the Colonial Nursing Association, which we reported last week, has been to quicken the interest of people on the other side of the Tweed in the excellent organisation which owes so much to the efforts of his wife. The Colonial Secretary very wisely avoided going into the details of the work of the Association, but appealed to the Scottish nation to support it on the great principle of obligation to the Empire. In his closing sentences he drew a vivid picture of the man employed in a distant colony by the Government struck down by illness with no woman of his own race or colour to attend upon him. This is the condition of things which the Colonial Nursing Association seeks to render impossible, and we know of no object which is more calculated to enlist the practical sympathy of the kind-hearted and charitable. Here it is not even easy to conceive such a state of things, for the tender ministrations of women are readily available. But it is our duty, as it should be our pleasure, to see that our kinsmen serving the State beyond the seas are able in their hour of necessity to enjoy, in their isolation from home and relatives, the attention which none but a woman of their own race can give. The Colonial Nursing Association is steadily making progress, but when the loftiness of its aim, and the need of its extension, are more fully realised, it will meet with a far greater measure of support than it has yet received.

### A REMINISCENCE OF MISS CURETON.

THE amount of the cheque presented in a purse to Miss Cureton, the former matron of Addenbrooke's Hospital, Cambridge, was £822 2s., and this and many other presentations were made to her last week. We congratulate the promoters of the testimonial on the sum subscribed, but handsome as it is, it scarcely reflects a tithe of the popularity which Miss Cureton enjoyed during the whole of her long term of office. One of her probationers, who has since had a good deal of experience at home and abroad, gave a charming description of her the other day. Describing her as an ideal matron, she said that "matron" used to go through the wards each morning at half-past seven, bidding "good morning" to every person as she went, and if by chance a probationer was absent and missed the greeting she felt that there was something wanting for the remainder of the day. Her kindness of heart, continued the narrator, was such that on one occasion when "a little pro.," fresh from home, hurt her leg rather badly during the first three weeks of her hospital experience, she managed to find time to bring her sewing into the bedroom and chat for half-an-hour at a stretch until the patient was about again. No wonder "the little pro." loved her.

### THE WAR NURSES.

THE *Orotava* arrived at Southampton from South Africa on the 31st ult. with the following nurses on board : L. M. Wright, A.N.S. (requires one month's leave and returns to South Africa) ; R. Woollcombe, A.N.S.R (wishes to return to South Africa at once) ; B. Turner, A.N.S.R. (resigns Army duty) ; A. Bron (Belgian nurse, one month's leave and returns to South Africa) ; M. P. Richardson (Canadian N.S., one month's leave and returns to South Africa). The following are "Invalids": A. Wishart, A.N.S.R. (requires two months' sick leave) ; F. M. C. Lindsay, A.N.S.R. (requires three months' sick leave) ; J. Wilson, volunteer (requires sick leave). The *Orcana* arrived at Southampton from South Africa on Tuesday ; the following are detailed as "ship's staff" : — Nursing Sisters E. M. Hayden, A. E. Gillam, B. Shapley, M. C. Meeke (all A.N.S.R., and all wishing to return to South Africa at once) ; M. E. Brooke-Smith and C. G. Jeffrey (A.N.S.R., requiring two and four months' leave respectively, and both returning to New Zealand). Nursing Sister L. Dawson, A.N.S.R., was invalided home.

### A NURSES' HOME WANTED AT A LONDON HOSPITAL.

THE interview between our Commissioner and the matron of the West London Hospital, reported in another column, shows that considerable progress in nursing, as in other respects, has been made in recent years at an institution which some years ago lagged behind. But the West London is still without sufficient accommodation for its steadily increasing nursing staff. As the matron says, more nurses will be needed when the new ward is opened, and we do not quite see where, under present conditions, they are to be put. In the temporary home the sisters have separate bed-rooms, but the probationers "sleep two in a room." Of course it is a question of money, and the matron would be only too delighted to be able to give every nurse a separate room. But as the committee still have ground at their disposal which can be built upon, we hope they will not lose much time in making provision for the nurses on a line with the general improvements in the hospital.

### THE SUPERINTENDENT NURSE OF SKIPTON UNION INFIRMARY.

IT will be seen from the letter of the Clerk to the Skipton Board of Guardians, which we publish in another column, that the superintendent nurse of the workhouse infirmary has been entirely and unequivocally exonerated from the charge of want of sympathy which was preferred against her by Colonel Maude, and, according to the *Yorkshire Daily Post*, endorsed by the board. On the strength of that report we naturally made, in our issue of October 26th, some severe comments on the conduct imputed to the nurse, and we are delighted to learn that she in no way merited them. We, of course, withdraw them with much pleasure, and regret that the action of the Skipton Board of Guardians, as reported in the daily press, led us into making strictures which it is now clear were wholly undeserved.

### A MISTAKE IN DISCIPLINE AT NEWHAVEN.

It is difficult to understand the position taken up with regard to the superintendent nurse of Newhaven Workhouse Infirmary by the majority of the Newhaven Board of Guardians. They have refused to assent to a motion to rescind a resolution passed at a meeting in September asking the superintendent nurse to resign, and yet they do not seem to have any serious cause of complaint against her. According to Mr. Mason "there was no dereliction of duty on her part, but the state of things was such that the master's authority was in danger." Mr. Willett, who also opposed the rescission of the resolution, said that "so far as the duties of a nurse went the Guardians had every confidence in Miss Emily Careless and they would be able to give her a testimonial. They only believed that she had made a mistake in discipline." This mistake appears to have been the failure of the superintendent nurse to report a disturbance to the Master. But, surely, the penalty exacted is utterly out of proportion to the offence. Here we have another instance of the abiding difficulty in regard to nursing in workhouse infirmaries. One may have the position of superintendent nurse, but in every petty detail the will of the master is supreme, and the interests and traditions both of the Clerks and Boards are generally in favour of supporting at all hazards the discipline of the house as impersonated in its master. What hope is there for the sick poor so long at this position is maintained?

### DINING IN A BATH-ROOM.

The Yarmouth Board of Guardians have tardily decided to instruct the architect to prepare plans for a nurses' home, separate from the workhouse. At the last meeting of the Board, the disclosures made were of such a character as to render action imperative. It was complained that, under present conditions, some of the nurses' bedrooms lead out of the lavatories, that others are placed over the kitchen, and immediately below the hot-water apparatus. A second complaint was that the night nurses have to dine in a brick-floored bathroom, in which there is no table, the edge of the bath being used for meals. Yet another serious indictment was that lights have to be burned day and night, as the place is infested with rats and mice. In fact, one of the guardians roundly declared that "the tramps off the road are ten times better provided for in a common lodging-house than the nurses in Yarmouth Workhouse Infirmary." We cannot understand, however, why no attempt has been made before to improve the nurses' abominable quarters.

### THE UNIFORM QUESTION AT DUNDEE.

The question of uniform has been giving trouble at the Dundee Poorhouse Hospital. A new uniform, which seems to have been decided upon for the sake of a hygienic material, was ordered by the authorities to take the place of the old. To this, it appears, no objection was taken by the nurses; but they asked that they might continue to wear the old uniform in the morning and the new in the afternoon. The request did not meet with favour, and the guardians have insisted upon the old uniform being entirely discarded. It is obvious that they were within their rights, but the settlement of the matter might fitly have been left to the discretion of the superintendent nurse.

### BRISTOL INFIRMARY.

A correspondent who has been studying the account in our columns of the East Dulwich Infirmary —now more correctly called the Southwark Infirmary—writes to suggest that a copy of The Hospital containing it should be sent to each of the Bristol Guardians, as they might then improve existing conditions for the probationers, both by arranging for an outside doctor to examine them in addition to the resident medical officer, and also by providing some means of recreation for their nurses, who, at present, she states, "are not even allowed a penny newspaper among them." We are afraid that the resources of the Bristol Guardians will not permit them to do things on quite such a liberal a scale as the St. Saviour's Board, but they might manage to afford a penny paper.

### THE NIGHT DIETARY.

The correspondent who last week asked "Why are night nurses neglected?" has elicited an interesting rejoinder from a matron whom, without any breach of confidence, we may describe as the head of a large staff in a very important city hospital. She herself has had the advantage of being a night nurse, and while she expresses the opinion that nurses nowadays are more difficult to please than of yore— an opinion fortified by an illustration from her own experience—she recognises the claims of the night nurses to every possible consideration. One difficulty in their case is, she points out, that of getting a sufficient variety of a dietary during the night. Her practical suggestion is that the night dietary of some of the hospitals should be published, and discussion invited. She adds, "I will gladly send ours if asked." We with pleasure act on the suggestion, and invite the matrons of some of our representative hospitals and infirmaries to send us their night dietary for the purpose of publication. In this way they may help other matrons who are sometimes at a loss to give sufficient variety, and do something to improve matters all round. They may also perhaps convince some nurses that they might "go further and fare worse."

### WORTHING ISOLATION HOSPITAL.

The advocates of false economy have failed at Worthing. Notwithstanding the recommendation of the Medical Officer of Health, that a permanent trained nurse at a salary of £60 a year should be appointed for Swandean Isolation Hospital, some of the members of the Worthing Town Council attempted to prevent it on the ground that she might not be wanted always. Happily, the contention of those who supported the appointment on the ground that it would tend to give the public greater confidence in the institution was successful. Fourteen members of the Town Council voted in favour of it, as against four who were hostile. With what possible confidence could a mother send a sick child to an isolation hospital which had not a trained nurse ready to receive the patient? The ratepayers of Worthing will have cause to be thankful if at times there is nothing much to do for the occupant of the post; but an isolation hospital without a nurse in charge is an absurdity.

### THE QUESTION OF TRAINING AT WATERFORD.

The question whether a particular hospital in Ireland with less than 140 beds should be recognised by the Irish Local Government Board as a training school may be arguable. But a member of the Waterford Board of Guardians is not content to plead that the conditions of nursing in the workhouse infirmary are such as to warrant the Local Government Board in recognising it as a training school. He goes on to contend that the nurses trained there are superior to the nurses trained in clinical hospitals with 140 beds. "With us," he says, "the nurses have to attend the surgeon in all his operations, while in large clinical hospitals this is all done by clinical students, and the nurses are merely attendants on the students. Consequently they cannot be as well trained as they are with us." The inference is that if this guardian could have his way the Local Government Board would regard the large clinical hospitals as inadequate training schools and the small workhouse infirmaries as adequate. We are not surprised that the chairman of the Waterford Board observed, "I cannot follow Mr. Smith. I may be wrong, but my idea is that girls cannot become proper nurses without going through all the different stages of hospital work in the right hospitals," and the subject dropped.

### GUARDIANS AND DISTRICT NURSES.

The Northampton Board of Guardians have shown their appreciation of the work of the Northampton Town and County Nursing Institution by increasing the subscription from 21 to 40 guineas. The chairman, who brought the matter before the board, said that 42 patients were on the books of the nursing institution at the present time, 13 of whom were in receipt of relief; and he spoke in warm terms of the excellent results of the labours of the lady superintendent and her staff. Another guardian observed that he believed the attention of the nurses to their cases helped to keep down the rates, and no opposition was offered to the augmentation of the contribution. In pursuing this policy of encouragement we are satisfied that the Northampton guardians are acting in the interests of the town, and we should be glad to see a larger number of boards equally liberal in their attitude to district nursing organisations.

### A NURSE'S VISIT TO A WORKHOUSE.

A few days ago a nurse was asked to inspect one of the large unions in the Eastern Counties. It happened that the day fixed was one of its regular board days, and therefore, no doubt, everything was made spick and span. She was kindly received by the master and mistress, or matron as she is called, and was immediately taken round the building accompanied by several of the guardians. The kitchens she thought disgraceful, and, in fact, on entering she went so far as to remark, "I suppose this is the laundry." She was then informed that new kitchens would shortly be finished. The little chapel was excellent and well looked after. The married peoples' quarters were all that could be desired and their comforts were studied in many ways. In one a happy pair were sitting by a tiny fire, the woman sewing for the house and the man chopping a few sticks, and a little kitten purring on the hearth. The wards were very empty, containing only a few patients with old ulcerated legs and bad eyes. The nurse in charge complained that she "got no interesting cases whatever, save in the labour wards." The saddest sight of all was in the men's department. Many were being kept because they were too lazy to work outside. Fighting, wrangling, and quarrelling were heard on all sides, and the work they were compelled to do seemed to be either done badly or not at all. The children were at play in the grounds. The last place visited was a small ward with old women in groups gathered together. Some were occupied, whilst others, very infirm, were sitting quietly, knowing well that it was their eventide and that soon they would pass away and be no more seen.

### THE NEW MATRON OF THE LONDON TEMPERANCE HOSPITAL.

The regret which will be generally felt in the nursing world at the resignation of Miss Lucas, matron of the London Temperance Hospital, who is retiring from hospital life, will be tempered by satisfaction at the announcement that she is succeeded by Miss A. T. Richardson, senior sister. Miss Richardson was trained at the London Temperance Hospital, under the régime of Miss Orme, and was lately presented with the South African War medal for services rendered at the front. She has the hearty good wishes not only of all connected with the hospital of which she is now matron, but of a wide circle of friends, for success in her new *rôle*.

### A SUCCESSFUL FIRST YEAR.

The first report of the Peckham Nursing Association more than justified the confidence with which the promoters set about their work. It was stated at the first annual meeting of the Council that, after meeting every expense, there remained a balance of £54 19s. 6d. to carry forward. This shows that the Peckham people appreciate the organisation, but the committee do well not to regard it as a reason for relaxing their exertions to secure further subscribers. Last year the nurse belonging to the Association paid 1,403 visits; and as the population of the district who are likely to require the aid of a nurse is rapidly increasing, it may soon become necessary to engage a second.

### SHORT ITEMS.

With regard to the free lectures to nurses at the City Orthopædic Hospital, Mr. Noble Smith will be followed on Fridays, November 8th and 22nd, by Mr. John Poland, whose subject will be "The Human Skeleton." Mr. Chisholm Williams will then lecture on "The Heart and Circulation," and "The Stomach and Digestion," while Mr Jackson Clarke starts the new year with "The Preparation of Patients for Operation," and "The Nurse's Duties in the Operating Theatre." This will complete the first half of the series.—Miss E. Mumford, one of the nurses who has just been dispatched to the Orange River Colony by the Colonial Office, was trained at the Women's Hospital, Sheffield, and has been on the staff of the Walsall Hospital for five years. She is the second nurse who has gone from the Walsall Hospital to South Africa.

### Lectures to Nurses on Medicine and Medical Nursing.

By Norman Dalton, M.D. London, F.R.C.P., Physician to King's College Hospital.

### XXIII.—COMA.

By "coma" is meant unconsciousness. The causes of this condition are, first, certain organic diseases of the brain, such as the rupture of an artery in it (whereby the blood pours out and tears up and compresses the brain substance), or a tumour, or abscess, or an injury (such as follows fracture of the base of the skull), or the late stage of meningitis, etc. In most of these cases the coma appears to be due to the compression of the brain substance between the bones of the skull and the blood clot, tumour, etc. Secondly, coma may be due to a poison in the blood. This poison may be generated within the body, as in uræmia, diabetes, and the coma which occurs in typhoid or other specific fevers; or it may be due to a poison introduced into the body from without, as in the coma of opium and alcoholic poisoning. If the blood be suddenly cut off from the brain, coma sets in, as may be observed in an ordinary fainting fit. Unconsciousness is also present in an epileptic fit, but we know little about the true nature of epilepsy. Coma may be preceded by delirium, as we frequently observe in typhoid fever. In many cases (whether preceded by delirium or not) it comes on gradually. In typhoid fever it may be preceded by simple obtuseness of intellect for two or three days, and it is curious to note that in many cases one of the last things that the patient can do before he lapses into absolute unconsciousness is to protrude his tongue when asked to do so. Nurses must remember, however, that in typhoid the patient often becomes deaf, and she must not mistake deafness for unconsciousness. In diabetes, uræmia, and other cases, it may take some hours for unconsciousness to become complete. When a small artery in the brain breaks, so that the blood leaks slowly out of the blood-vessel, unconsciousness may not come on for some minutes; but if a large artery breaks and the blood rushes forth, complete coma may be established almost instantaneously. Now it is necessary for a nurse to know something about coma, because the symptoms which accompany its onset are of extreme value to the doctor in enabling him to find out the cause of the coma, so that the nurse must know what to look for and must write down what she observes as soon as she can.

The coma which accompanies a hæmorrhage into the brain is so associated with disturbances of the motor functions that it will be treated of in a lecture by itself. The coma which is due to intoxication with alcohol may be so profound that the patient cannot be roused at all. The face is usually flushed, the breathing stertorous (that is, noisy and snoring), the pulse rapid and full, and the pupils "equal" and, very likely, dilated. The breath may smell of alcohol, but as people who are feeling ill on account of a slowly leaking cerebral hæmorrhage may have taken some brandy before they became unconscious, too much importance must not be attached to this symptom. If the clothing be loosened, so that the neck and chest are free, patients can mostly be left alone until the intoxication has passed off. There is a slight risk, however, of food getting into the windpipe during the act of vomiting and producing suffocation. If the dose of alcohol be exceptionally large, there is a risk of collapse accompanying the coma, in which case the pulse becomes weak and the face pale. In such a case, depressing remedies, such as cold douching, which are often used in alcoholic coma, are injurious. Warmth should be applied and the doctor summoned, who will, probably, inject strychnia hypodermically, and take other measures to restore the heart's power.

In "opium" poisoning the patient becomes slowly, but eventually, completely unconscious. The breathing is slow and stertorous, and the pupils are both contracted to pin-points. The skin is moist and pale, and towards the end becomes cyanotic. The pulse is quick and feeble. If the patient can swallow, he should be given a cup of strong, black coffee, and should be kept awake, if possible, by walking him up and down the room, or by slapping or flicking the skin. A teaspoonful of Condy's fluid in a tumbler of water can also be given. The rest of the treatment must be carried out by the doctor, but the nurse may give a tablespoonful of mustard in a tumblerful of warm water as an emetic as soon as she sees the case. It must be remembered that in alcoholic and opium poisoning, and in poisoning by chloral and other drugs which produce unconsciousness, the stomach pump is the best means of getting rid of such of the poison as remains in the stomach, for it can be used not only to empty the stomach but to wash it out.

As regards the coma which is produced by poisons which are generated in the body, uræmic coma has been described with Bright's disease. Diabetes is a disease in which a large quantity of grape sugar is found in the urine. But the essential point is that the blood is heavily charged with sugar, and the patient becomes emaciated and extremely weak. In addition, he suffers from thirst, an excessive flow of urine, dryness and roughness of the skin, various skin eruptions, such as boils or carbuncles, and many other symptoms. It may be as well to mention, however, that in women there is often great irritation and even eczema at the external genitals, in consequence of the passage of the sugary urine, and this symptom has often led to the discovery of the disease. Now a large number of cases of diabetes die in a state of coma. The coma is often brought on by some extra fatigue, such as a long walk or a long railway journey, or by the occurrence of some other illness (such as influenza, etc.) or of one of the complications of diabetes, such as carbuncle, gangrene, etc. It is also said that it is liable to occur if all sugar and all starchy food is suddenly cut off from the patient's diet, a method of treatment which is essential but should be carried out gradually. Nurses must particularly guard diabetics from fatigue. The "coma" comes on gradually, and is usually preceded by incoherence of ideas or delirium. It slowly becomes complete, and in a typical case the breathing is very characteristic, namely the respirations are full, deep, and as rapid as is compatible with their depth and fulness. This mode of breathing is described in the lecture on the respiration. The breath has a sweetish smell. The pulse is quick and weak, the skin is cold, and the temperature subnormal. Nothing can be done for these patients, though they have appeared to improve on injecting large quantities of salt solution into the veins.

Nurses are not called upon to diagnose cases of coma; but, as they may have to supply facts to the doctor, it may be of use for them to know that in cases of coma due to poisoning, the pupils, whether dilated or contracted, are equal in size, that if any convulsions or paralysis affect the muscles, these symptoms nearly always affect both sides of the face and limbs; and, lastly, that the coma always comes on gradually. On the other hand, when coma is due to lesions in the brain, the pupils are often (not always) unequal, the convulsions or paralysis are usually seen on one side only, and unconsciousness "may" come on instantaneously. It cannot be too often repeated that unconscious patients must be fed with the greatest care to prevent food getting into the windpipe, and that great attention must be paid to the bladder. They often do not pass water until great distension of the bladder is produced, and then it may dribble away into the bed, producing the idea that the bladder is emptying itself, while, as a fact, it is over full to a dangerous extent.

### The Nurses of the West London Hospital.

A CHAT WITH THE MATRON.

By Our Commissioner.

Started in the interests of the locality, the West London Hospital has developed into a general institution of very considerable importance; though its accommodation has lately been extended, and will be further augmented shortly, even then it may be doubted whether it will suffice for the fast-growing wants of the rapidly-increasing number of the working classes in the far west of the metropolis. Its progress during the last twenty years is attested by the number of nurses at the existing time, as Miss Hardy, the matron, told me on the occasion of my visit last week.

"The West London Hospital only became a training school when I came here twenty years ago, and there are now 46 nurses, including seven sisters."

"Has the increase been gradual?" I asked.

"Yes; we started with one or two. But when the new ward is opened next year we shall require another sister and six more nurses."

"The course is for three years?"

"It has been so for the last six years. It was originally 18 months, and then two and a half years. At the end of this three years' training the probationers can stay as assistant nurses, if they choose. Four of the sisters were formerly probationers; but, as a rule, they do not remain. There is no settled number of assistant nurses."

"What salary do you offer to assistant nurses?"

"From £20 to £30. The probationers are not paid any salary the first year, but they receive £15 the second, and £20 the third year."

PAYING PROBATIONERS.

"Have you any paying probationers?"

"Yes; ladies can come here and work with the nurses for three months on payment of 15 guineas. They are at liberty to take their meals in the hospital, but not to sleep in it. Of course, we do not give them any certificate. They obtain a little experience which is useful in the home, but if they want to adopt nursing as a profession, it is of no value to them. Some of them are helpful in the wards, and others are quite the reverse. The committee think it is a fair arrangement that the ordinary probationers should not receive payment during the first year, as the training is exceptionally excellent, owing to the absence of students."

THE POST-GRADUATES AND NURSES.

"The post-graduates do not prejudicially affect the position of the nurses?"

"No. The post-graduates do no dressings. They simply go round with the visiting staff, and, in fact, they rather tend to make things better for the nurses, because the medical staff lecture to the post-graduates over the beds and the nurses hear what the doctors are saying. If they keep their ears open, they can pick up a great deal."

"I suppose that they also have the usual instruction?"

"They have two courses of lectures, medical and surgical, during each of the three years, and at the end of each course there is an examination."

"The certificates," continued the matron, "are not given until they leave the hospital. That is to say, if a probationer stays on as an assistant nurse, she does not get her certificate until she leaves."

What does she do, then, if she is seeking another post?"

"I write and state that the certificate will be given her at the termination of her engagement. We never place any obstacle in the way of probationers leaving at the end of their three years, but it is a rule not to sign it until they go. However, as I have already intimated, most of them do go as soon as their term is up."

AGE AND UNIFORM.

"At what age do you take probationers?"

"Twenty-two. The committee fixed that age; it is quite young enough. As to the limit, we do not as a rule accept a candidate who is more than 30, but if she has been in a children's hospital, or the circumstances are very special, we may make an exception."

"I see that you require an entrance fee of two guineas. Is it returnable?"

"No, it is intended to cover the board and lodging for the month the probationer is on trial. Some who come for the month are found to be unsuitable, and others find that they do not like the work."

"And the uniform?"

"That is provided by the committee at the cost of the probationer. We get the caps made, and keep the material for the uniform, acting, in fact, as the shop. Uniform is not compulsory out of doors, but the nurses usually wear it for preference, because it is so easy to put on over their cloaks."

"Do you have as many applications as you require from girls wishing to become probationers?"

"More than we require. A certain number to whom I send the details never reply, because they are either too young, or too old, or do not like the conditions. But I always have a long list in hand from which to choose, though, of course, I find that some have gone elsewhere when I want them."

DUTY AND OFF DUTY.

"What is the number of probationers to each ward?"

"In each of the larger wards there is a sister and four probationers; and in the smaller wards a sister over two wards and two probationers in each. At night there are two probationers in each of the large wards, and one probationer in each of the smaller. There is a sister in charge of all the wards. In any special cases we put on an extra nurse. There is one permanent nurse in the theatre, and each sister takes in with her case a probationer to wash out the sponges. A sister and two probationers are attached to the out-patient department and do all the casualty work."

"The hours of duty are another important question?"

"The probationers breakfast at 7, go on duty at half-past, and go off at 8 P.M. They are allowed an hour out during the day, and they may be out in the evening until 9.30. One day every week they are off-duty from 5 P.M. They have also a whole day off every month, and on Sunday, either in the morning, afternoon, or evening, they get church time. The sisters have 'a long Sunday' and 'a short Sunday' alternate weeks—church time one week, and half a day the next. As to holidays, the probationers have a fortnight, the assistant nurses two or three days over, and the sisters three weeks."

NO RELIGIOUS TEST.

"Do you often have to enforce the misconduct clause?"

"Oh, no. We have a very nice class of nurses, steady and well-behaved. But the clause is there in case of need."

"You mention Church time. Is there any rule as to attendance at any particular church?"

"On the contrary, we are quite unsectarian. Provided that the nurses obey the rules, they may attend what church or chapel they please. We have no chapel in the hospital, and the committee, as well as the nurses, belong to all religions. The prayers in the morning are such as any can join in."

"Is anything done in the hospital in the way of providing recreation for the nurses?"

"We used to have a very nice lawn for tennis, but it is now taken up by the new wing and the post-graduates. The patients have still grounds, and go out a good deal. The nurses may cycle when they are off duty, but as there is no side entrance, they have to keep their machines at a cottage close by."

THE WANT OF MONEY.

"Is there a pension scheme in connection with the institution?"

"Several of the staff are members of the Royal National Pension Fund, but the hospital cannot afford to help the nurses in the matter of pensions. There is no settled income for the purpose. All that can be done is to make a presentation to a nurse who has been here a long time when she leaves. There was recently a presentation of this kind by the committee."

"Lastly, how are you off in respect to accommodation for the nurses?"

"We have only a temporary nurses' home, which adjoins the hospital. The sisters have separate bedrooms, but the probationers sleep two in a room. Each sister has a sitting-room attached to her ward, and the probationers a general sitting-room in the home. There is a large general dining-room in the hospital. The accommodation is fair, if homely, and we do all we can to provide for the happiness and comfort of the nurses. When we get money enough, we shall have a permanent home. The committee would do a great deal if only they had the means; but it is absolutely essential to practise economy."

### The Nurse's Never.

BY A MEDICAL LECTURER ON NURSING.

(*Concluded from page* 60.)

Never take the temperature in the armpit until you are sure the skin is dry.

Never neglect to chart the temperature as soon as you have taken it.

Never allow the patient to take the temperature himself. Many patients are more knowing than nurses where there is a question of temperature.

Never take the temperature in the rectum or vagina without first having oiled the thermometer.

Never say the temperature is "better" or "worse." State the figures. A temperature of 97° is lower than one of 105°, but it can hardly be termed better if the patient is dead.

Never use anything but a graduated measure for measuring doses of medicine, unless ordered to administer the dose in "drops."

Never pour out a dose of medicine anywhere but near the patient. A dose to the wrong patient or a double dose to the right one may not harm the patient, but it will certainly harm you when it is discovered.

Never put a hot-water bottle next to the skin. Its efficacy and the patient's safety are both enhanced by surrounding the bottle with flannel.

Never put india-rubber next the skin either with hot water or ice. The one may cause blistering, the other sloughing.

Never neglect the hot-water bottles or foot warmers in the early morning. This is the very time when they are most needed.

Never complain that you cannot get a feeding cup if there is a teapot to be had instead.

Never administer a quantity of food to a patient until you have found out if he can swallow.

Never reply that a patient has eaten "a good meal" or "very little." State the kind of food and the amount. To say that your charge ate "nothing yesterday and twice as much to-day" may amuse the doctor but does not enlighten him.

Never leave food by the patient's bedside.

Never do any work in a ward or sick-room during the meal hour.

Never give a patient a cup that has overflowed into the saucer.

Never disregard a patient's intelligent craving for particular articles of diet.

Never allow a patient in bed to use a cold bed-pan or urinal.

Never put strong carbolic acid into a bed-pan or anywhere where a patient's skin is likely to come in contact with it.

Never allow excreta to remain in the sick room.

Never throw away vomited matter until it has been examined by the doctor.

Never give a patient a medicated bath without having a clear notion of the materials to be employed, and the strength of them.

Never use your patient as a thermometer for estimating the temperature of the bath. Although he turns red in hot water and blue in cold, the record is not exact, and there are other objections of a more or less obvious nature.

Never allow a patient to be waked out of his first sleep either intentionally or accidentally.

Never imagine that a patient who sleeps during the day will not sleep during the night. The more he sleeps the better will he be able to sleep.

Never employ a heavy hand when touching a patient. Quickness, lightness and gentleness are quite compatible.

Never hurry or bustle.

Never stand and fidget when a sick person is talking to you. Sit down.

Never sit where your patient cannot see you.

Never require a patient to repeat a message or request. Attend at once.

Never speak to a patient from behind, nor from the door, nor from any distance from him, nor when he is doing anything.

Never speak to a patient suddenly, nor give him food suddenly, especially if he is inattentive.

Never speak to a patient who is standing or moving unless you know he can bear the strain. "Standing is, of all positions, the most trying to a weak patient."

Never do anything in a patient's room after you have put him up for the night.

Never judge the condition of your patient from his appearance during a conversation. See how he looks an hour afterwards.

Never be undecided with your patients. "Let your doubt be to yourself, your decision to them."

Never read aloud to patients unless they are children, eye-patients, or uneducated. People who like to be read to have generally not much the matter with them.

Never read a story to children if you can tell it.

Never read fast to a sick person. The way to make a story seem short is to tell it slowly.

Never play the piano to a sick person if you can play on strings or sing.

Never confine a patient to one room if you can obtain the use of two.

Never disregard the patient's "fancies." Study them, they signify something important or instructive.

Never allow monotony in anything.

Never allow too much variety.

### Everybody's Opinion.

[Correspondence on all subjects is invited, but we cannot in any way be responsible for the opinions expressed by our correspondents. No communication can be entertained if the name and address of the correspondent are not given, as a guarantee of good faith but not necessarily for publication. All correspondents should write on one side of the paper only.]

### "FLIRTATION AND COSTUME."

"Charles Woodbridge," clerk to the Uxbridge Joint Hospital Board, writes under date of October 31st: The attention of this Board has been called to a paragraph headed as above in The Hospital of October 19th inst., and I am desired by the Board, in justice to the matron, to inform you that the paragraph referred to is incorrect and misleading.

[Mr. Woodbridge omits to say in what respect the "Note" he refers to is either "incorrect" or "misleading." The statement on which we commented had appeared in both the local and the London press; and, as we understand, has been a subject of conversation among the Guardians.
Ed. The Hospital.]

### NURSES AND THE CORONATION.

"One of the Public" writes: I have been waiting to see whether the proposal that nurses should be specially provided with seats to witness the Coronation procession would appear to any of your readers to be a little unreasonable. I am aware that when it was put forward last month you made a kindly response, though not making any promise. But it seems to me that in this instance nurses can hardly expect that they should be shown any favour which cannot be shown to the members of any other profession. I have the utmost admiration for those who are engaged in the work of nursing, and they deserve all that is said in praise of their self-denial and zeal. Yet, I fancy sometimes that they run the danger of being spoilt by an excess of consideration. Why should they not, like the rest of the community, take their chance of seeing the Coronation procession? Of course I am quite ignorant as to whether a large number of them are willing to subscribe a sum sufficient to ensure them the reservation of seats along the route. But even that is a matter for them to arrange. I think it is not for their advantage that they should be treated like children for whom someone else has to make provision.

### A SISTER ON THE R.A.M.C.

"An Invalided Orderly" writes: I read with much attention Sister Green's opinion of the R.A.M.C. Having been in the R.A.M.C. for two years as a "Civilian Compounder," her somewhat original ideas interest me greatly. As there are "black sheep in every flock," so, in the R.A.M.C. we have ne'er-do-wells. But why should the whole corps be "run down" on account of these few? As to the conduct of the orderlies towards "sisters," it has always seemed a pity to me that sisters and orderlies could not work together. Are they not both working in the same cause? I should not like to say on which side the fault lies, but I have personally experienced several cases where the fault was on the side of the sisters. They are sometimes too apt to look upon orderlies as "errand-boys" or "washers-up," and it is only natural that the orderlies should strongly resent such treatment. Orderlies are still human beings and have feelings. Because he has to do the rough part of the work, must an orderly be looked down upon by the sisters who once upon a time were "pros." themselves, and had then to do the dirty work? Of course there are many sisters who take a kindly interest in their orderlies, and I venture to remark that they do not regret it.

### WHY ARE NIGHT NURSES NEGLECTED?

"A Matron who was once a Night Nurse" writes:—I am much interested in the letter about the night nurses, and think there is a great deal that is true in it. The case of the night nurses and the arrangement of their food is always a great anxiety to every conscientious matron, but I am bound to say that if "Sympathiser" had been a matron, and had faced the many difficulties of the post, she would have been more merciful in her judgment. Nurses nowadays are more difficult to please than of yore. For instance, one nurse could eat neither sausages, herrings, nor eggs, yet grumbled because a fourth thing was not provided, which probably she would also have refused. I know quite well that during my own term of night duty my appetite was not as good as on day duty, but I always had enough, and the food in those days "*was* rough." It is very difficult to get sufficient variety for dietary during the night, and I think the mistake in many institutions is in the poor tired night sister presiding at the night nurses' morning meal, instead of either the matron or the home sister. The night sister should be left to have her own dinner in peace without having to carve, and she cannot complain so easily if she dines herself—the home sister can speak for others if she presides. In large institutions it is often impossible for a matron to preside, but if she can, it is most necessary, as the housekeeper, however kind, has not been trained, and cannot sympathise with the poor tired night nurses. May I suggest that the night dietaries of some hospitals be published, and discussion invited? I will gladly send ours if asked.

### THE SKIPTON UNION INFIRMARY.

"M. R. Knowles," Clerk to the Skipton Board of Guardians, writes under date of November 1st: The attention of the Guardians of the Skipton Union has been drawn to certain reports of the meeting held on the 12th ult., which have appeared in your and other journals relative to the alleged inhumanity on the part of the superintendent nurse of the Skipton Union Infirmary. The board (through the medium of the house committee) have gone fully into the matter and very much regret that such reports, in many respects untrue and exaggerated, and calculated to be prejudicial to the welfare of the nurse in question, as well as create a false impression in the minds of the public, should have been allowed to be published. Mr. Bagenal, the Local Government Board inspector, was present at the meeting of the guardians on Saturday, the 26th ult., after interviewing the nurses, and after hearing the matter discussed, stated that he was naturally pained, as a great many other people were, on reading certain accounts which he was very glad to hear were not accurate or proper reports of what had really taken place. I am directed by the guardians to forward to you a copy of a resolution passed at their last meeting on Saturday the 26th ultimo, and to request that you will give the same publicity thereto, and to this letter, as was given to the report complained of.

The following is the resolution referred to:—

Skipton Union.

*Copy Resolution.*

October 26th, 1901.

"That, owing to an unfortunate combination of circumstances, Mr. Spink was not informed of his wife's critical condition, and that the Board hereby expresses its regret for the omission, and tenders him its sincere sympathy in his bereavement; at the same time, the Board has every confidence in its nursing staff, and does not think there has been any intentional want of sympathy on the part of any of its officers."

### THE NURSE'S NEVER.

"A. R." writes: I should like to say a few words on "The Nurse's Never." Seldom have we the opportunity of choosing our patient's bed; generally speaking, we are not sent for unless the patient be very ill, and if on a double bed, however great the desire may be on the nurse's part to have the patient on a single bed, we have to abide by circumstances and nurse on the double one. Then as to allowing the under or draw sheet to become "rucky," no nurse, even in her own interest, will allow it to be so. It is one of the first and most important lessons taught in the wards of hospitals, etc., and no nurse is likely to forget it. The

"square of carpet" on polished boards at the bedside can only be meant for the private nurse. Yet I was sent to a case where the patient had that very same each side of her bed, and polished boards, and proud she was of the former. We all know that neither a ward nor a sick-room is meant for a "skating rink." Also, my last patient would not on any account have her hair in plaits, but arranged on the top of her head or at the back, whichever she wished, and she was not the first or only one who did the same. What staff nurse or sister would allow her ward, or what private nurse her sick-room, to become a "drying shed"? "The Nurse's Never" cannot be meant for "probationers" or the hospital staff, so I presume the advice is meant for the private nurse. Moreover, what nurse could conform to those rules in private nursing without turning the house upside down, and either being sent away or disliked by the patient's friends whilst she was in the house? I, with other nurses, am looking forward to next week's HOSPITAL for the continuation of "The Nurse's Never."

### ON DISCIPLINE.

"A MATRON" writes: It has been suggested by an able and kindly writer that a "happy medium of discipline can be easily achieved under the auspices of the 'Ideal' Matron." Let me say a word in defence of our matron, that unfortunate lady of many responsibilities. Were the discipline of probationers to begin and end with the standard of perfection aimed at by the average capable matron, training would be a rather different matter, and fully-trained nurses would be rather different women. A matron's ideal nurse is thorough, trustworthy, conscientious, gentle, patient and capable, and it is the matron's desire that all in authority under her control (such as sisters and staff nurses) should train the raw probationer in those qualities which are best suited to the life of a good woman and a well-trained nurse. But (and this is my defence for the lady) the matron, unfortunately, is not directly respons ble, and that is where the shoe pinches in many hospitals. It is, of course, absolutely impossible that the head of a large nursing staff can be constantly in the wards watching the carrying out of her ideal discipline; her multifarious duties forbid such a thing, therefore to the sisters and staff nurses must necessarily be entrusted directly the training and discipline of the young probationers. The matron seldom, if ever, hears of the petty tyrannies, the nagging, the injustices which weary and fret the beginner. Some may say, "But a capable matron should know everything about her nursing staff." But it is not possible. Let the head be ever so vigilant, ever so active, ever so kindly intentioned, there is inevitably a chasm between her actual knowledge and what really takes place. What is required (and until we get it hospital life for juniors will continue to be a mixed pleasure) to achieve the happy medium of discipline is the ideal sister and the ideal staff nurse to conscientiously carry out the aims and wishes of the capable matron. There is no possibility of good discipline being instilled if a sister shows herself to be an unjust woman: once allow a probationer to feel convinced that she need not expect justice from the sister under whose authority she is, and all good influence is at an end, for the time at least. We have all heard the criticisms of our seniors, from our fellow probationers such as, "When I am a sister I shan't be so hard on my probationers as sister —— is;" or, "When I am a staff nurse, I shall not make the probationers do all the work, I shall do a little of it myself." Excellent aspirations, both of them; but, unfortunately, very seldom carried out. The coveted post of staff nurse is soon within our grasp, but what a change takes place when we don the uniform of this mighty position. We must sit at sister's table when she is off duty, and, of course, we cannot do this and help the probationers with the work; still the prick of conscience is quieted by the thought, "Oh! well I had to do all the work while my staff sat and did nothing, the pros. must just manage as I used to;" and another nail goes into the coffin of discipline because of the lack of conscience. When sisters maintain discipline with justice and reason, and when staff nurses teach discipline by conscientiousness and patience, then, indeed, we may hope that the ideal medium will be achieved, and the aims of the average capable matron be carried out.

### Appointments.

ATCHAM WORKHOUSE INFIRMARY.—Miss Betty Ann Richards has been appointed nurse. She was trained at Belper Union Workhouse Infirmary.

BLACKHEATH AND CHARLTON HOSPITAL.—Miss Alexander has been appointed matron. She was trained at Charing Cross Hospital, where she occupied the various posts of ward sister, theatre sister, and home sister, previous to her appointment as assistant matron of the Royal Infirmary, Sheffield.

CHELTENHAM UNION INFIRMARY.—Miss Marguerite E. Oakey has been appointed superintendent nurse. Since her training she has been sister for five years at the Worcester General Infirmary, and sister for two years at the Birmingham Infirmary. She holds the L.O.S.

COTTAGE HOSPITAL, PEMBROKE.—Miss P. J. Brady has been appointed nurse matron. She was trained at Paddington Infirmary, and has since been staff nurse, for eight years, at The Hospital, Stratford-on-Avon.

FARNHAM JOINT ISOLATION HOSPITAL.—Miss E. Berry has been appointed nurse. She was trained at St. William's Hospital, Rochester, and has since been charge nurse at the Isolation Hospital, Guildford, and charge nurse at Willesden Sanatorium.

HUNGERFORD UNION.—Miss Kate Dunnell has been appointed assistant nurse. She was trained by the Meath Workhouse Nursing Association at the Royal Hospital, Sheffield.

LONDON TEMPERANCE HOSPITAL.—Miss A. I. Richardson has been appointed matron. She was trained at the London Temperance Hospital for three years, where she has since been staff nurse and sister. She has lately served in South Africa as a member of the Army Nursing Service Reserve.

NORTHERN HOSPITAL, WINCHMORE HILL, N.—Miss Emily Mearns has been appointed superintendent of night nurses. She was trained at the Royal Sick Children's Hospital, Aberdeen, and has held the post of charge nurse at the Northern Hospital for nearly four years. Miss Susan Milne has been appointed housekeeper. She was trained at the North Riding Infirmary, Middlesbrough, and was for six years staff nurse and sister at Southwark Infirmary, and lately night superintendent at Poplar and Stepney Sick Asylum. Miss Marion Kirkwood has been appointed charge nurse. She was trained at Hackney Infirmary, Homerton. Miss Mabel Tolley has been appointed charge nurse. She was trained at The Birmingham Infirmary.

POPLAR AND STEPNEY SICK ASYLUM.—Miss Clara Marriott has been appointed night sister. She was trained at Poplar and Stepney Sick Asylum for three years, and has since been staff nurse there for one year and sister for two years.

ROYAL ALBERT EDWARD INFIRMARY, WIGAN.—Miss Agnes Warburton has been appointed nurse. She was trained at the Royal Infirmary, Liverpool, and since her training has been a member of the private nursing staff.

### Wants and Workers.

THE HOSPITAL.—Would any nurse like THE HOSPITAL posted Monday after publication? If so, please send three months' postage to Miss Slater, S. Andrew's House, Mortimer Street, London, W.

EAR TRUMPET.—District Nurse "Conway," Church Fields, Salisbury, would be very glad if someone would send her an ear trumpet for a very deaf patient whom she has to attend.

AIR CUSHION.—District Nurse would be glad if anyone having an air cushion or water pillow no longer in use would kindly give it for a bedridden patient greatly in need of one. Address, District Nurse, 36 Wire Street, Dresden, Longton.

### Presentations.

Addenbrooke's Hospital, Cambridge.—Miss Cureton, on her retirement from the post of matron of Addenbrooke's Hospital, received from the Weekly Board a handsome illuminated framed resolution of thanks and regret. The Mayor of Cambridge, Mr. H. M. Taylor, presented her with a testimonial consisting of a beautiful Russian leather silver-mounted purse (the gift of Mr. Adams and Miss Raynes) containing a cheque for £822 2s. subscribed to by friends, and past and present nurses of Addenbrooke's Hospital, and with the cheque a book in vellum and gold (the gift of Mr. J. S. Clay) containing an address from the mayor and list of more than 300 subscribers. Miss Cureton was also the recipient of many other handsome presents. From the honorary staff, a silver-mounted travelling bag; the sisters, silver tea caddy and spoon. The probationers, a large silver-mounted inkstand; the porters, servants, scrubbers, and laundresses, an electro-plated cake dish. The following is the text of the address:—

A Tribute of Respect to
Miss Mary Newcombe Cureton.

On her resignation and retirement after fourteen years' tenure of the post of matron of Addenbrooke's Hospital, a purse containing contributions from more than three hundred subscribers was presented to Miss Cureton as a token of esteem and affection, and in grateful recognition of her loving solicitude for the patients, her constant attention to the comfort and convenience of the other inmates of the hospital, and her loyal endeavours to meet the wishes of the governors and the staff.

Signed, on behalf of the subscribers,
H. M. Taylor,
Mayor, Chairman of Committee.

Cambridge, October, 1901.

London Temperance Hospital.—On Monday the vice-chairman of the London Temperance Hospital, Colonel Sheffield, presented the matron, Miss Lucas, with a gold bracelet, as a token of the appreciation of her services by the Board of Management on her retirement from hospital work. Miss Lucas has been engaged in nursing for some sixteen years, and has been associated with the London Temperance Hospital for the past eight years, first as home sister and later as matron. Colonel Sheffield said that the members of the board had very highly appreciated the manner in which she had discharged her services to the hospital, and although they had full confidence in Miss Richardson, the lady they had appointed in her place, yet it was with reluctance that they let her go. There were present the Rev. Dr. Burns, honorary secretary, Colonel Bolland, and other members of the board. The chairman, Mr. Alderman Strong, telegraphed his deep regret that his magisterial duties detained him elsewhere. Miss Lucas has received presents from the members of the nursing staff, the servants, and porters, and others connected with the hospital, all of whom unite in expressing their sense of loss at her leaving, and in wishing her a very pleasant holiday in the south of France, for which she started on Tuesday morning.

City Hospital, Walker Gate, Newcastle-on-Tyne.—On Monday, Miss B. N. Ogston, the matron of the City Hospital, Newcastle-on-Tyne, was presented by the "staff" with a gold brooch, set with diamonds and ruby, in commemoration of her twentieth year as matron of the institution.

### Where to Go.

British Home and Hospital for Incurables, Crown Lane, Streatham.—Sale of work, in the hall of the institution, Thursday, November 14th, to be opened at 3.30 p.m. by the Lady Mayoress.

Empress Rooms, Royal Palace Hotel, Kensington.—Bazaar in aid of the Building Fund of the Gordon Hospital, Vauxhall Bridge Road, S.W., Friday, November 8th, and Saturday, November 9th, from 2 30 to 10 p.m.

The Modern Gallery.—Mr. Herbert J. Finn is holding an Exhibition of Water-Colour Drawings of York Minster, Durham Cathedral, Oxford, and Dutch Sketches, at The Modern Gallery, 175 Bond Street, W. The Exhibition will be open from November 11th to December 28th inclusive.

### For Reading to the Sick.

SEND OUT THY LIGHT.

"Send out Thy Light!" the way is dark before me,
The path Thy Love has moulded out for me;
"Send out Thy Light!" that I may see Thy Footsteps
Tracking the waters of life's restless sea.

"Send out Thy Light!" the clouds are dark above me,
Gathering a tempest from the angry sea;
"Send out Thy Light!" that I may see the storm-drops
Falling from the dear Hand, once pierced for me.

"Send out Thy Light!" and lead me, Father, lead me
Beyond this darkness, sorrow and unrest;
"Send out Thy Light!" and guide me, worn and weary,
To the calm shelter of my Saviour's Breast!

*Beatrice E. Bishop.*

Pain and suffering grow holy when we think how through them the Father comes to His children. Let us not be cheated by mere theories to say that sorrow is not dreadful, sickness not wearisome, and bereavement not sad. We only mock the sufferers all round us when we say that. But let us claim that if a man really is close to God there is a victory over the pain, and a transfiguration of the sadness. "If a man is close to God." Can we say that, and not remember how the Godhead and the manhood met in the Incarnation? Can we say that, and not remember that all we have been saying was supremely realised when the Son of God was born and lived, and died for us? God's being! Who could doubt it, as he walked the streets, and men saw God in His face? He brought it with Him across the threshold of the Temple, and through the low doorway of the cottage of Bethany. God's pity! Who did not see it as He laid His hands upon the children's heads and looked down from the Mount of Olives on Jerusalem! God's truth! Who must not hear it speaking as He talks with Nicodemus, or preaches from the mountain? God's power! What more has it any need of proof, when the finger laid upon the hem of His garment gives the lost health back again, when the death upon the Cross is the salvation of the world? All that is consolatory in God—being, sympathy, truth, power—Christ has set in the clearness and the splendour of His life.

And so if you want consolation you must come to Him. It is not a dead phrase. It was not dead when He spoke it first in Jerusalem, and said, "Come to me." It was the very word of life. You must come to Him, know Him, love Him, serve Him. In His Church and His service you must take your place. Nay let us not say "must." Our duties are always best stated as our privileges. You may come to Him, for He has said, "Come unto Me all ye that are weary and heavy laden, and I will give you rest." May we all come nearer and nearer to Him always, and find peace.

*Phillips Brooks.*

I leave thee never; thou art not alone
And with thine own, and thee, thine angels dwell
Possess thy soul in patience; freely give
Me love for love, and all shall yet be well.

The time is short. They that now weep ere long
Shall be as though they wept not; they that mourn
Be comforted, for I will comfort them;
And sweet shall be their glad thanksgiving song.

*From "Lyna Mystica."*

### Echoes from the Outside World.

### AN OPEN LETTER TO A HOSPITAL NURSE.

There seems only a slight hope that most of our soldiers will be able this Christmas to enjoy the pipe of peace, but Queen Alexandra has determined that some 6,000 of the men shall, at any rate, smoke the pipe of remembrance. The pipes Her Majesty contemplates sending are to be good briar ones, silver mounted, and upon the mount will be stamped a crown and the Queen's monogram. The makers are required to deliver them in sufficient time to allow of their reaching South Africa before Christmas, and the orders have only been placed with the manufacturers upon condition that this can be done without fail. It is not exactly stated upon what grounds the selection will be made, but that they are "for presentation to men of the regiments with which the Queen is directly connected." It is true that when Queen Victoria sent out chocolate two years ago, she distributed boxes to all her soldiers alike. But at that time the number of troops serving in South Africa was comparatively small, whereas now it is immense, and to give to all would involve an enormous outlay. Let those who are sorry that all will not receive a pipe contribute as much as they can to the Christmas funds now on hand, and they will then feel that they, as well as Her Majesty, are doing their utmost to brighten the Christmas of the men who are serving their country abroad.

---

Although few of us were able to put our words into verse as did the Poet Laureate in his latest poem—

"Welcome, right welcome home, to these blest Isles,
Where, unforgotten, loved Victoria sleeps"—

yet in our own way we all rejoiced on Saturday that the Duke and Duchess of Cornwall and York had returned to us in safety. Seven months of continuous travel over sea and land is a long time, and it is a matter for real thankfulness that during all that period so little untoward occurred—only one death amongst the 577 persons who set sail in the *Ophir*, whilst on the other hand, as the Duke said himself, they had "gained a great, pleasant, and profitable experience, and made many friends." The weather on the morning of the home coming was beautiful and in striking contrast to the gusty cloudy days which had come earlier in the week, and also to the dense fogs which have since set in. All the harbour, and every corner which might afford a chance of a view was black with people, and right away from Portsmouth to London the sides of the railway were more or less thronged with loyal subjects waiting to welcome and to cheer. Those who stood expectant were in most cases well rewarded. The windows of the saloon carriages are large and wide and a good deal could be observed beside the rose-decked engine and the white-washed coal in the tender. The King remained for some time without his hat, and thus allowed a good view of his face to be seen. The Duchess with her newly-recovered treasures about her, in company with the Queen, sat close to the window pointing out anything of interest to the little lads as the train rushed by.

---

But, of course, it was the passing of the procession to Marlborough House which attracted most attention, and thousands were grouped along the mile and a half between the Royal residence and Victoria Station. At Hyde Park Corner, I am told, not one-tenth of the mass congregated saw anything, but in Grosvenor Place there was no crush, in some places the sightseers being only two deep. Great was the pleasure of most to note the little grandson, Prince Edward, in the first carriage with the King, and the extreme gravity of his demeanour, as he punctiliously acknowledged the universal enthusiasm, was exceedingly comic. The King, in his warm overcoat, I rejoiced to see, looked not only happy in mind but well in body; but I could not help wondering whether it was wise even for a Sailor Prince who looked so thoroughly "fit" as the Duke of Cornwall to drive in the keen wind with no great coat on because the sun was shining. The Duchess looked cosy in her Canadian sables, and, of course, the last carriage with manly Prince Albert, pretty Princess Victoria with the dear curly head, and bonnie baby Prince Henry, came in for a vast amount of attention. I fear that the first sample of weather London had to show on the Sunday was hardly to the taste of the travellers who have been to climes where fogs are unknown, but how little they cared for such details was evident on Monday when the whole party drove in the open to St. Pancras on their way to Sandringham so as to be seen of all beholders. And this when the mist was yellow and thick.

---

The King must have grieved at the bad news from South Africa which cast a shadow on the universal rejoicing. The engagement at Bakenlaagte was disastrous as far as our loss is concerned, though the wonderful courage and endurance of the little body of men can only arouse our admiration. They were attacked in a blinding rainstorm by a thousand Boers, and though they never saw the enemy till they were closely surrounded, rallied at once around the guns. Colonel Benson was soon shot down, and Major Woole-Sampson took his place. All day, though men and officers fell quickly, the stubborn resistance went on, till at nightfall Colonel Barter brought assistance. The English lost nearly three hundred men, the Boers about the same. Colonel Benson only lived till the fighting had ceased.

---

The marriage of Mr. Herbert Gladstone on Saturday was a matter of interest to many who had never seen either the bride or the bridegroom, because of the historic name he bears. Not that he himself is unknown in the world of politics, but it is an interesting fact that whilst Mr. Herbert Gladstone is a Liberal, the father of Miss Dorothy Mary Paget represented various divisions of Somerset in Parliament for 30 years uninterruptedly as a Conservative, so that two families, who for years have held different political views, have joined hands in matrimony. The bridegroom was especially rich in clerical relatives; his cousin, the Bishop of Rochester, tied the nuptial knot, assisted by two brothers-in-law and a brother. The bride was beautifully gowned in a dress of white silk gauze over silver tissue embroidered in silver and opals, the Court train being of old lace hung from the shoulders, and it was carried by two little pages in herald's costumes of red velvet. The seven bridesmaids—the eighth was unavoidably absent at the last moment—included Miss Dorothy Drew. They also wore dresses of white silk gauze, but over pink Oriental satin, embroidered with bands of silver and opals, and they carried loose bunches of Madonna lilies tied with satin ribbon. They wore no hats but tulle veils, and wreaths of flowering myrtle—an old fashion which is again finding much favour. Amongst the gifts was a large silver inkstand from the King, bearing the Gladstone crest, and inscribed, "To the Right Hon. Herbert John Gladstone, M.P., on the occasion of his marriage, from Edward R. and I., 2nd November, 1901." The jewellery given to the bride by her future husband included an emerald half hoop which the late Mrs. Gladstone had left for her son Herbert's bride, should he marry, which at one time seemed improbable. The presents numbered altogether upwards of 700.

### Notes and Queries.

**The Editor is always willing to answer in this column, without any fee, all reasonable questions, as soon as possible.**

**But the following rules must be carefully observed:—**

**1. Every communication must be accompanied by the name and address of the writer.**

**2. The question must always bear upon nursing, directly or indirectly.**

**If an answer is required by letter a fee of half-a-crown must be enclosed with the note containing the inquiry.**

---

*Duties of a District Nurse.*

(43) Can anyone tell me if a district nurse in the country is expected to perform the domestic duties of cleaning her patient's house and cooking the food?—*Domestic.*

A district nurse is not concerned with the domestic needs of her patient—that is, however, required from the cottage nurse, though a nurse frequently does something more than her duty in some cases.

*Manual on Nursing Children.*

(44) Is there a nursing manual, bearing particularly on children's diseases, which I could recommend to my probationers?—*M. McN.*

"The Mother's Help and Guide to the Domestic Management of her Children," by P. Murray Braidwood, M.D., price 2s., would be useful.

*Nurse on Liner.*

(45) A nurse would be glad to learn how she may obtain a post as nurse (not stewardess) on the large Atlantic liners.—*J. E. T.*

At present none of the large Atlantic liners carry nurses as part of their staff.

*Jamaica.*

(46) Will you kindly tell me if there are any hospitals or nursing homes in Jamaica; and where to apply?—*Nurse S.*

Apply the Matron, the Public Hospital, Jamaica.

*Quarantine.*

(47) Will you kindly tell me where a nurse could go for quarantine in London after attending a case of scarlet fever?—*E. J. H.*

The Nurses' Hostel, Francis Street, W.C., makes provision for this.

*Home for Paralysed.*

(48) Would you kindly tell me of a hospital where a middle-aged man, mentally afflicted with paralysis of the nerves, could be taken? He could afford to pay 8s. a week at present.—*S. S.*

The National Hospital for the paralysed and Epileptic, Queen's Square, Bloomsbury, W.C.

*Light Employment.*

(49) Could you kindly tell me how a well-educated gentleman could obtain home employment? He is suffering from rheumatoid arthritis, which has affected his hands. I should also be extremely grateful to anyone who would give a wheel chair to the same patient, or would take in exchange a hot-air bath.—*M. J.*

We fear we can give no useful suggestion, so much depends on capacity. Possibly he could manage to undertake book-keeping for his local tradesmen, or do some private teaching.

*Lost Certificate.*

(50) What would you advise a nurse who has lost her three-year certificate to do? Is it possible to obtain a duplicate?—*Nurse M.*

Your training school would possibly give you a duplicate, or act as referee.

*Memorial Nurse.*

(51) What is a Memorial Nurse?—*E. R. E.*

We presume a nurse maintained in memory of some valued life.

*Hygienic Clothing.*

(52) Will you kindly tell me if Dr. Lehmann's Reform Cotton Wool underclothing is as hygienic as woven wool, and less liable to give a chill?—*C. E. J.*

Cotton will never equal wool in certain respects, but openly woven cotton fabrics are very useful in their way, and stand washing better than woollen goods.

*Hair Shirt.*

(53) Can you tell me where I can obtain what is called a "hair shirt" to promote the action of the skin and restore impaired circulation of the blood?—*R. B.*

Possibly Messrs. Jaeger can help you. Rough flannel might answer the purpose.

*Etiquette.*

(54) 1. Has a nurse, with a Queen Charlotte's certificate, as good a social standing as a nurse with general training? 2. Is it etiquette for nurses to advertise for work, and to have a plate on the door?—*Pug.*

It depends upon the social position of the nurse before she was trained. The general training is the higher professional qualification. 2. Yes; certainly.

*Prison Nursing.*

(55) Where can I apply for information as to position of nurse in prisons?—*Media.*

The Prison Commissioners, Home Office, Whitehall, London, S.W.

*Canada.*

(56) Can you tell me if Ontario, Canada, would be a good place for a married district nurse, whose husband is incapacitated from work, to settle in? Any information gratefully received. 2. Would there be any difficulty in obtaining another post as district nurse in England, where my husband would be allowed to share my cottage?—*Irish Nurse.*

Write to the Emigration Information Office, 31 Broadway, Westminster, for latest returns of nursing prospects in Canada. 2. Try; see advertisements and advertise.

*Training.*

(57) 1. Is the Salford Royal Hospital a recognised training school for nurses? 2. What does the word "recognised" mean in this connection? 3. Are nurses trained in poor law infirmaries eligible for as good posts as those trained in general hospitals?—*Naomi.*

1. Yes. 2. It means that the school in question has complied with the conditions laid down by the Local Government Board, and that the nurses trained by it are "recognised" as eligible for the post of superintendent nurse in poor law institutions. 3. Yes, though certain individual schools have a better reputation than others.

1. Does the Cleveland Street Infirmary's certificate rank with those of the principal London hospitals; and would it be sufficient for private nursing? 2. What kind of cases are nursed in such an infirmary?—*M. W. L.*

The Central London Sick Asylum, Cleveland Street, W., and Hendon, is one of the larger recognised training schools, and the three years' certificate granted there constitutes a full nursing qualification. 2. All kinds of non-infectious cases.

*Army Nursing.*

(58) Will you kindly give me some information as to how I may train for the Army Nursing Service? Is there any institution I could join?—*K. F.*

See the "Nursing Profession: How and Where to Train," for full details. You must have three years' training in a civil general hospital first. Then, upon being accepted by the military authorities, you will be required to undergo six months' probation at Netley.

*Defective Eyesight.*

(59) 1. Can you give me the name and address of the foremost practical oculist in London or the provinces? 2. Also can you tell me if anything can be done to cure spots which permanently obstruct the vision, or whether they are likely to prove fatal to the eyesight?—*C. A.*

1. We never give names. 2. You must consult a specialist.

*Sister.*

(60) Kindly let me know whether a nurse, holding the Medico-Psychological Society's certificate only, and who is now a charge nurse in an imbecile ward, is entitled to be addressed sister?—*Sister.*

She is entitled to be called what the superintendent nurse arranges.

*Ear Tubes.*

(61) I should be glad to know where I can obtain the long-ear tube which used to be sold at the Soho Bazaar at 2s.—*M.L.P.*

Probably any of the surgical instrument makers could procure them for you.

*British Columbia.*

(62) Will you kindly give me the address of your correspondent in Victoria, British Columbia, *re* maternity nursing in that colony?—*No Name.*

We do not give the addresses of our correspondents. Address your letter to the care of the Editor, enclosing stamps, to forward communication.

---

**Standard Books of Reference.**

"The Nursing Profession: How and Where to Train." 2s. net; post free 2s. 4d.
"Burdett's Official Nursing Directory." 3s. net; post free, 3s. 4d.
"Burdett's Hospitals and Charities." 5s.
"The Nurses' Dictionary of Medical Terms." 2s.
"Burdett's Series of Nursing Text-Books." 1s. each.
"A Handbook for Nurses." (Illustrated). 5s.
"Nursing: Its Theory and Practice." New Edition. 3s. 6d.
"Helps in Sickness and to Health." Fifteenth Thousand. 5s.
"The Physiological Feeding of Infants." 1s.
"The Physiological Nursery Chart." 1s.; post free, 1s. 3d.
"Hospital Expenditure: The Commissariat." 2s. 6d.
All these are published by the Scientific Press, Ltd., and may be obtained through any bookseller or direct from the publishers 28 and 29 Southampton Street, London, W.C.

### Travel Notes.

BY OUR TRAVELLING CORRESPONDENT.

### LXXXV.—IN THE VALLEY OF THE VAR.

In the exquisite and numerous valleys running towards the interior from the Riviera, there is such a wealth of exquisite scenery, interesting architecture, and beauty of almost all kinds that the difficulty is to make a selection amid such an *embarras de richesses.* To-day I think I shall speak of

### TOUET-LE-BEUIL, PUGET-THÉNIERS, ETC.

These places can be reached direct in a day's excursion from Grasse *viâ* Colomars, or from Nice. The two roads are equally beautiful. If from Nice, you pass, before arriving at Colomars, numerous charming little villages, such as La Madeleine S. Isidore, where a glimpse of S. Jeannet is seen and the beautiful villages of Carozza and Le Broc are visible in the far distance. After Colomars the scenery is still finer, and when the Var is a rushing torrent instead of a dry water-course it must be magnificent. One of the most striking points is near to the Pont Charles Albert where, at a dizzy height above the foot of the gorge clings the rock village of St. Germaine; the train stops immediately underneath, but so great is the altitude that the church appears no bigger than a diminutive toy. Touet-le-Beuil is a delightful place; what a hunting field for the photographer and artist. I perched myself precariously on a rock commanding a view of the church, and the innumerable rich brown and red roofs that constitute the village; nobody caught sight of me and I sketched in comfort. The church is very singular; it is built over a strong and lofty arch which spans a rushing torrent, leaping down on its way to join the Var. Inside the church a grating affords one a view of this torrent which is foaming along only a few feet below. You cannot comfortably do Puget-Théniers and Touet-le-Beuil in one day. I should recommend for one day's outing that you should take train to Touet and cycle back, because, being all slightly down hill, it makes a most agreeable run of about 33 miles.

Stop at La Vésubie, where the scenery is grand, the gorge becomes very narrow, and the mountains seem to frown threateningly over you, causing a feeling of general personal insignificance.

### PUGET-THÉNIERS—GUILLAUMES AND ENTRAUNES.

Puget-Théniers has drawn upon itself some severe remarks concerning dirt and smells even from such a hardened traveller as Mr. Hare, and Mr. Potter, the author of a delightful little cycling guide that was the joy of my existence on the Riviera, sounds his warning trumpet to the same tune. He says: "Puget-Théniers is undoubtedly the dirtiest and most malodorous village I have ever visited . . . the friend who was with me tersely summed up the place as a 'beastly hole,' and I cannot say that this description is not an accurate one."

But in spite of a considerable amount of dirt and odours far removed from the gales of Araby, I enjoyed the place; probably were it cleaner and sweeter it would not be half so picturesque. I made a sketch in a corner that could hardly be surpassed, I think, in dirt, neither could it be rivalled in rich colour and picturesque decay. The houses, balconied and richly carved in wood, were hung with ancient garments probably of small value from any but an artistic point of view, but what soft and yet vivid hues these admirable rags spread before my admiring gaze. The elegant leisure of Puget-Théniers surrounded me with astonished interest, scratched its elbows in contemplation, and was lost in joyful recognition of neighbours' garments as they beheld them transferred from the fairy-like balconies to my prosaic canvas. I do not think that it is a place to stay in, though I have often taken up my residence in far worse, but it is very pretty and bright, and one can see it all and go as far as Guillaume all in one day.

The Knights Templars once had a holding here, and the present place occupies the site of their garden. It is now bright and lively with much washing going on round the fountain and innumerable gay awnings, and every kind of yellow and red diligence add colour to the scene. If you take the first train from Nice it will give you time to go to Guillaume and thence to Entraunes; if you are cyclists you will be independent of the diligence entirely, but to feeble walkers it is a great help and will take you from Puget to Guillaume in four hours. The place itself is nothing remarkable, nor is Entraunes, but the road through the gorge of the Daluis is worth traversing. A very pretty view of Puget may be had by crossing the bridge and climbing the opposite hill, which in the month of March was covered with primroses and a very small and charming white flower wholly unknown to me.

One more place to be seen close to Puget is Entrevaux. It is distant four miles, and I believe an omnibus goes there but I made the journey on foot. Puget being the present terminus of the Sud railway in that direction, is quite an important little place, and causes the old garden of the Templars to resound with diligence bells.

### ST. MARTIN LANTOSQUE.

This is a suitable place to stay in, and alas! is becoming fashionable; when one hears that there is English Church service one knows what to expect. It is used chiefly as a summer resort from Nice, and has no less than three hotels. If you are going only for a week end from Nice or Grasse, take train to Plan-du-Var station and thence to St. Martin, 23 miles, a lovely drive.

It is an admirable centre for excursions, and if you can manage it I would recommend a week to be spent there.

There is the romantic walk along the bridle-path to Entraque, this takes eight hours, and a mule and guide are necessary, but it is a superb excursion. A mile and a half from St. Martin, up the Vésubie, you come to the stone that marks the boundary, and stepping beyond it, you are in Italy. A mule path (20 miles) takes you to the Baths of Valdieri, an unimportant place, useful only as a deeper centre for still more secluded excursions. There is a way to Turin from St. Martin Lantosque *via* Entraque and Cuneo, but in these days of hurried travelling and "doing" a country in a fortnight, such leisurely ways are not contemplated.

Next week I shall tell you of one or two places that can only be seen by the assistance of a cycle, a mule, or on one's own legs. There are many such on and about the Riviera, and but little known to the touring public. The distances are long, but very often you can get a lift in a train by a little management, and walking is easy in the fresh mountain air. I am far from being a first-class walker, but I can do almost anything about which I tell you, though failing, I must admit on those pleasant occasions when the cycle has to be carried on one's back. I think none who have not tried it can form any idea of the loveliness of these regions from March to the end of May.

### TRAVEL NOTES AND QUERIES.

RULES IN REGARD TO CORRESPONDENCE FOR THIS SECTION.— All questioners must use a pseudonym for publication, but the communication must also bear the writer's own name and address as well, which will be regarded as confidential. All such communications to be addressed "Travel Editor, 'Nursing Mirror,' 28 Southampton Street, Strand." No charge will be made for inserting and answering questions in the inquiry column, and all will be answered in rotation as space permits. If an answer by letter is required, a stamped and addressed envelope must be enclosed, together with 2s. 6d., which fee will be devoted to the objects of "The Hospital" Convalescent Fund. Any inquiries reaching the office after Monday cannot be answered in "The Hospital" of the current week.

HARROGATE BOARDING HOUSE (Miss H.).—A correspondent kindly sends me the following address. Miss Garth, Spring Vale Villa, 46 Walker Road, Harrogate. It is close to Baths and Wells.

PRIVATE NURSING IN FLORENCE (Florence Abroad).—Second-class single via Newhaven, Dieppe and Milan £5 8s. 7d. Third-class (quite possible), £3 12s. 10d. Clothing should be woollen of varying thicknesses, but never be without it for under garments. Florence is often very cold and even in hot weather the changes are very great from the sun to the shade. No luggage is allowed free on Italian railways except what you can take with you in the carriage. The expense of it will be about 15s. through to Florence, if you are moderate in your ideas. Read my articles on the subject of what to take and what to leave in "The Hospital" for December 24th, 1898.

